# Editorial: Involvement of Bioactive Peptides in the Control of Cell Survival, Proliferation and Plasticity in Physiological and Pathological Conditions

**DOI:** 10.3389/fendo.2021.767733

**Published:** 2021-09-22

**Authors:** David Vaudry, Dora Reglodi

**Affiliations:** ^1^Normandie Univ, UNIROUEN, Inserm U1239, Laboratory of Neuronal and Neuroendocrine Communication and Differentiation, Neuropeptides, Neuronal Death and Cell Plasticity Team, Rouen, France; ^2^Normandie Univ, UNIROUEN, Inserm, Regional Cell Imaging Platform of Normandy (PRIMACEN), Institute for Research and Innovation in Biomedicine (IRIB), Rouen, France; ^3^Department of Anatomy, University of Pecs, Medical School, PTE-MTA PACAP Research Team and Szentagothai Research Center, University of Pecs, Pecs, Hungary

**Keywords:** neuropeptides, cell survival, proliferation, plasticity, bioactive peptides, peptides, physiology, pathology

The scope of this Research Topic is to highlight the involvement of bioactive peptides in the control of cell survival, proliferation, and plasticity in physiological and pathological settings. Bioactive peptides are small amino acid chains that act on target effector cells and their sources can be diverse. As illustrated here, bioactive peptides include neuropeptides (PACAP, oxytocin, substance P, ODN), gastrointestinal peptides (secretin, VIP, ghrelin, galanin, gastrin), plant peptides (caryophyllaceae-type cyclopeptides), and fragments of proteins (FAM19A5, cerebrolysin). But wherever they come from and whatever the functions they were discovered for, it appears that a growing number of peptides can promote neuronal survival and plasticity. For example, secretin was initially identified for its gastrointestinal functions but also promotes cell survival in the hippocampus and the cerebellum during development and regulates neuronal plasticity and memory in these two brain regions in adulthood (Wang and Zhang). Other peptides such as oxytocin, known to stimulate milk ejection and uterine contraction, or ODN, known for its involvement in stressful behavior or food intake, are now the subject of studies for their ability to reshape neural circuitry or to promote neuroprotection (Pekarek et al.; Masmoudi-Kouki et al.), paving the way for therapeutic applications for the treatment of neurodevelopmental, neuropsychiatric and neurodegenerative diseases. Some researchers are looking for the potential role of other peptides, such as ghrelin in neurodegenerative disease (Kim et al.), and putative neuropeptides, such as FAM19A5, for the treatment of traumatic brain injury (Shahapal et al.). This suggests that we may still discover further bioactive peptides controlling cell survival, proliferation, and plasticity.

For some peptides such as PACAP, the neuroprotective activity seems to be well established (1) to the point of looking for the most efficient administration routes to reduce the volume of the infarct lesion and to promote functional recovery after stroke (Cherait et al.). However, Maugeri et al. highlight rightly that this peptide often has contradictory effects on neuronal survival depending on the tissue or the duration of the treatment. For instance, in the early phase of amyotrophic lateral sclerosis (ALS) progression, PACAP promotes motor neuron survival and axonal regeneration, whereas at later stages of the disease it promotes neuro-inflammation responsible for motor neuron degeneration (Maugeri et al.). PACAP is thought to protect cells through autocrine or paracrine mechanisms, but the interpretation of its action can be complicated by expression levels of the peptide and its receptors that vary depending on the cell type and the time after injury. While in ALS patients PAC1R is downregulated (Maugeri et al.), Woodley et al. show that PAC1R, VPAC1R, and VPAC2R are up-regulated for at least 2 weeks in the distal part of the sciatic nerve after transection. PACAP and VIP expression are also upregulated with a peak of expression after 2 and 7 days, respectively. Based on these kinetics of expression and functional studies, the authors conclude that PACAP could promote the expression of early proinflammatory mediators required to attract macrophages to the injured sciatic nerve, while VIP would prevent excessive macrophage recruitment and stop the inflammatory response at later stages. Opposite effects of PACAP and VIP are also reported by Takeuchi et al. on mouse cortical neurons (Takeuchi et al.). Indeed, while PACAP promotes neurite outgrowth, VIP impaires axon outgrowth and decreases dendrite arborization through activation of the VPAC2 receptor. Taken together, these results show to what extent a peptidergic system can be efficient but also extremely complex. On top of that, peptides can act alone but also as a cocktail as shown by Kang et al. with cerebrolysin, which decreases hippocampal neuronal death and increases brain-derived neurotrophic factor (BDNF) expression after one week of administration in a pilocarpine-induced seizure model. The fact that neuropeptides probably work together in a complementary or synergistic manner to fine-tune cell survival, proliferation, or plasticity is an idea taken up in several studies of this Research Topic (Zeng et al.; Wang and Zhang; Takeuchi et al.). As mentioned in those manuscripts, this is supported by recent studies showing co-expression of neuropeptide receptors capable of forming heterodimers leading to a complex pharmacology. Therefore, in future projects, it will not be sufficient to study one particular neuropeptide, and we will have to focus on groups of neuropeptides co-expressed in cells or released sequentially.

Besides acting on neurons, bioactive peptides can also control cell survival, proliferation, and plasticity in peripheral tissues. This is the case of PACAP, which exerts numerous protective effects on peripheral organs such as the airways, liver, heart, kidney, and intestine (Toth et al.). If PACAP could, among others, be a useful tool for the treatment of osteoarthritis formation, this is also the case of substance P, which promotes chondrocyte proliferation and differentiation (Li et al.). All these effects of neuropeptides and some of their similarities of actions in physiological or pathological conditions illustrate the complexity of the regulations involved and the therapeutic potential offered by these molecules when we are able to understand their subtleties.

Endogenous neuropeptides and their receptors are often overexpressed in cancer cells, prompting scientists to consider them as therapeutic targets. This is the case with galanin, the expression of which is found in most brain tumors (Falkenstetter et al.). This could be of interest for the treatment of these cancers since on other cell types, galanin exerts anti-proliferative effects and promotes apoptosis. Exogenous bioactive peptides such as cyclopeptides from plants are also tested for their cytotoxic activities on tumor cell lines (Houshdar Tehrani et al.). This suggests their possible use for cancer treatment, but in view of the numerous effects that certain peptides can have on the organism, it will be necessary to carry out in-depth studies to exclude the existence of undesirable effects at the central or peripheral level. To conclude, the most difficult issue is probably not to find functions of interest for a bioactive peptide, but to succeed to find the relevant clinical application.

## Author Contributions

DV and DR have co-chaired this Research Topic. DV and DR wrote together this editorial. All authors contributed to the article and approved the submitted version.

## Funding

This work was supported by INSERM (U1239), Rouen University, Pecs University, Normandy Region and the European Union. Europe gets involved in Normandy with European Regional Development Fund (ERDF). 

## Conflict of Interest

The authors declare that the research was conducted in the absence of any commercial or financial relationships that could be construed as a potential conflict of interest.

## Publisher’s Note

All claims expressed in this article are solely those of the authors and do not necessarily represent those of their affiliated organizations, or those of the publisher, the editors and the reviewers. Any product that may be evaluated in this article, or claim that may be made by its manufacturer, is not guaranteed or endorsed by the publisher.
